# Delayed Diagnosis of Common Variable Immunodeficiency Revealed by Recurrent Respiratory Infections and Persistent Mediastinal Lymphadenopathy: A Case Report

**DOI:** 10.7759/cureus.113296

**Published:** 2026-07-24

**Authors:** Mohammed Aharmim, Mohamed Lakhal, Nezha Reguig, Loudiyi Sara, Jamal Eddine Bourkadi

**Affiliations:** 1 Pulmonology and Phthisiology, Moulay Youssef Hospital, Rabat, MAR; 2 Respiratory Diseases, Faculty of Medicine and Pharmacy, Centre Hospitalier Universitaire (CHU) Mohammed VI, Mohammed I University, Oujda, MAR; 3 Pulmonology, Faculty of Medicine and Pharmacy, Moulay Youssef Hospital, Mohammed V University, Rabat, MAR; 4 Pulmonology, Moulay Youssef Hospital, Centre Hospitalo Universitaire (CHU) Ibn Sina, Rabat, MAR; 5 Pulmonology, Moulay Youssef Hospital, Rabat, MAR

**Keywords:** common variable immunodeficiency, hypogammaglobulinemia, iatrogenic hypogammaglobulinemia, mediastinal lymphadenopathy, primary immunodeficiency

## Abstract

Common variable immunodeficiency (CVID) is the most common symptomatic primary immunodeficiency in adults and is characterized by impaired antibody production, hypogammaglobulinemia, and recurrent infections. Owing to its marked clinical heterogeneity, the diagnosis is frequently delayed, particularly in patients presenting with atypical manifestations.

We report the case of a 57-year-old woman who presented with recurrent lower respiratory tract infections associated with persistent mediastinal lymphadenopathy. Initial chest computed tomography revealed a right-sided pneumonic consolidation with centrilobular tree-in-bud micronodules. Follow-up imaging demonstrated complete resolution of the pulmonary micronodules but persistent mediastinal lymphadenopathy, prompting extensive investigations. Histopathological examination of an axillary lymph node showed reactive follicular hyperplasia, while microbiological investigations, including Xpert MTB/RIF and bronchoalveolar lavage cultures, were negative. Further evaluation excluded sarcoidosis, hematological malignancy, and other infectious causes. Serum protein electrophoresis revealed marked hypogammaglobulinemia, and quantitative immunoglobulin testing demonstrated markedly decreased serum IgG (0.98 g/L; reference range: 7-16 g/L) and complete IgA deficiency (0 g/L; reference range: 0.7-4.0 g/L), establishing the diagnosis of CVID.

This case highlights the diagnostic challenge posed by late-presenting CVID and emphasizes that recurrent respiratory infections associated with persistent lymphadenopathy should prompt consideration of an underlying primary immunodeficiency, even in older adults. Early recognition and timely initiation of immunoglobulin replacement therapy are essential to prevent irreversible pulmonary complications and improve long-term outcomes.

## Introduction

Common variable immunodeficiency (CVID) is the most common symptomatic primary antibody deficiency in adults and is characterized by hypogammaglobulinemia, impaired antibody production, and recurrent bacterial infections [1,2]. It comprises a heterogeneous group of disorders with variable clinical manifestations, ranging from recurrent infections to autoimmune diseases, granulomatous inflammation, lymphoproliferative disorders, and malignancies, making the diagnosis particularly challenging [1,3].

Although CVID is a primary immunodeficiency, its diagnosis is frequently delayed because of its heterogeneous presentation and the absence of specific clinical features during the early stages of the disease. Previous studies have reported a median diagnostic delay ranging from approximately four to nine years, a delay associated with an increased risk of irreversible organ damage, chronic pulmonary complications, and immune dysregulation [4,5]. Most patients are diagnosed between the second and fourth decades of life; however, some individuals remain undiagnosed until late adulthood, particularly when recurrent infections are initially considered isolated events rather than manifestations of an underlying immune disorder [2,4,5].

Respiratory tract involvement is the most common clinical manifestation of CVID and represents the leading cause of morbidity and mortality. Recurrent sinopulmonary infections, chronic bronchitis, pneumonia, bronchiectasis, pulmonary nodules, and granulomatous-lymphocytic interstitial lung disease are frequently encountered [1,4,6]. In addition to infectious complications, approximately one-third of patients develop non-infectious manifestations, including persistent lymphadenopathy, splenomegaly, autoimmune diseases, and granulomatous lesions that may mimic sarcoidosis, tuberculosis, or lymphoproliferative disorders, often leading to extensive investigations before the diagnosis of CVID is established [3,7,8].

Persistent generalized lymphadenopathy is a recognized but often misleading manifestation of CVID. Histopathological examination usually reveals reactive follicular hyperplasia or benign lymphoid proliferation, making the exclusion of lymphoma and chronic infections mandatory [7,8].

We report a case of delayed diagnosis of CVID in a 57-year-old woman presenting with recurrent respiratory infections associated with persistent mediastinal lymphadenopathy. This case highlights the importance of considering CVID in adults presenting with recurrent respiratory infections and unexplained persistent lymphadenopathy, even in the absence of a previous history suggestive of immunodeficiency.

## Case presentation

A 57-year-old woman with no significant past medical history was referred to our pulmonology department for recurrent lower respiratory tract infections evolving over the preceding year. She presented with a chronic productive cough associated with mucoid sputum, recurrent episodes of purulent expectoration, and intermittent fever. Several courses of antibiotics resulted in only partial and transient clinical improvement. She denied weight loss, arthralgia, ocular symptoms, skin lesions, or other systemic manifestations.

**Figure 1:**
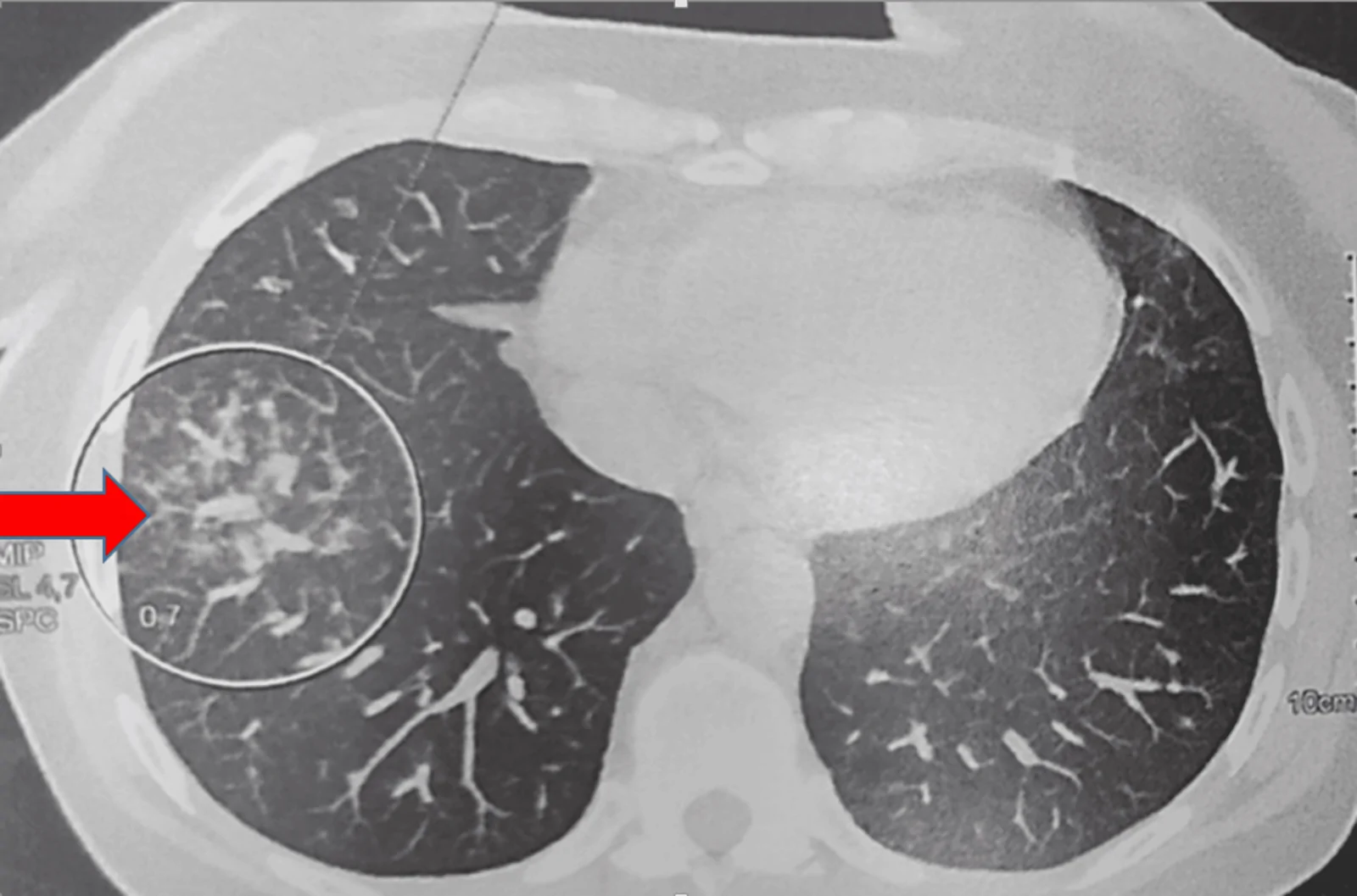
Initial chest computed tomography scan (2024) Right-sided pneumonic consolidation associated with centrilobular tree-in-bud micronodules (red arrow)

An initial chest computed tomography (CT) scan performed in 2024 revealed a right-sided pneumonic consolidation associated with centrilobular tree-in-bud micronodules (Figure 1).

**Figure 2:**
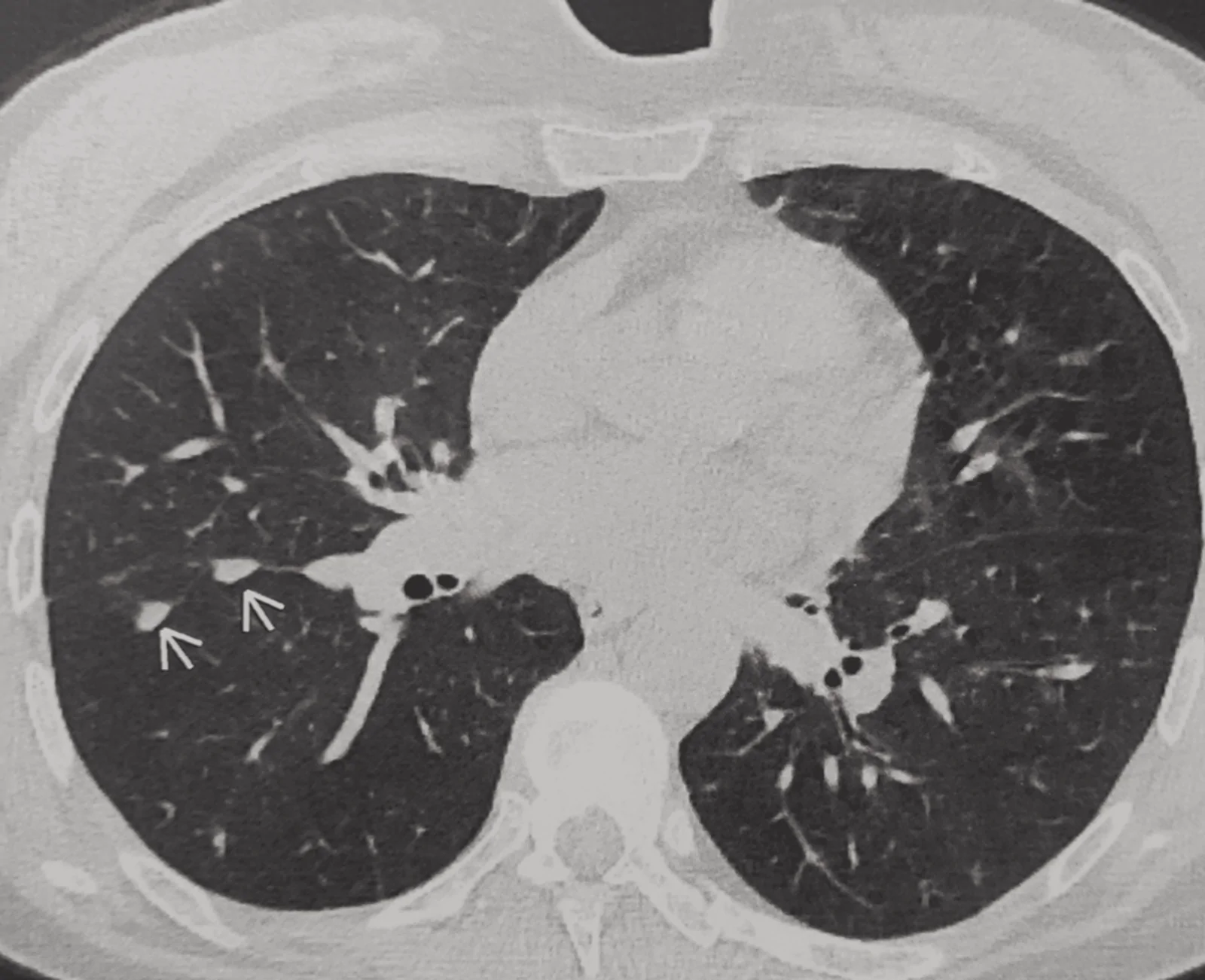
Follow-up chest computed tomography (CT) scan (2025, lung window) Complete resolution of the tree-in-bud micronodules following antibiotic therapy, with the appearance of two small nodules in the right upper lobe

**Figure 3:**
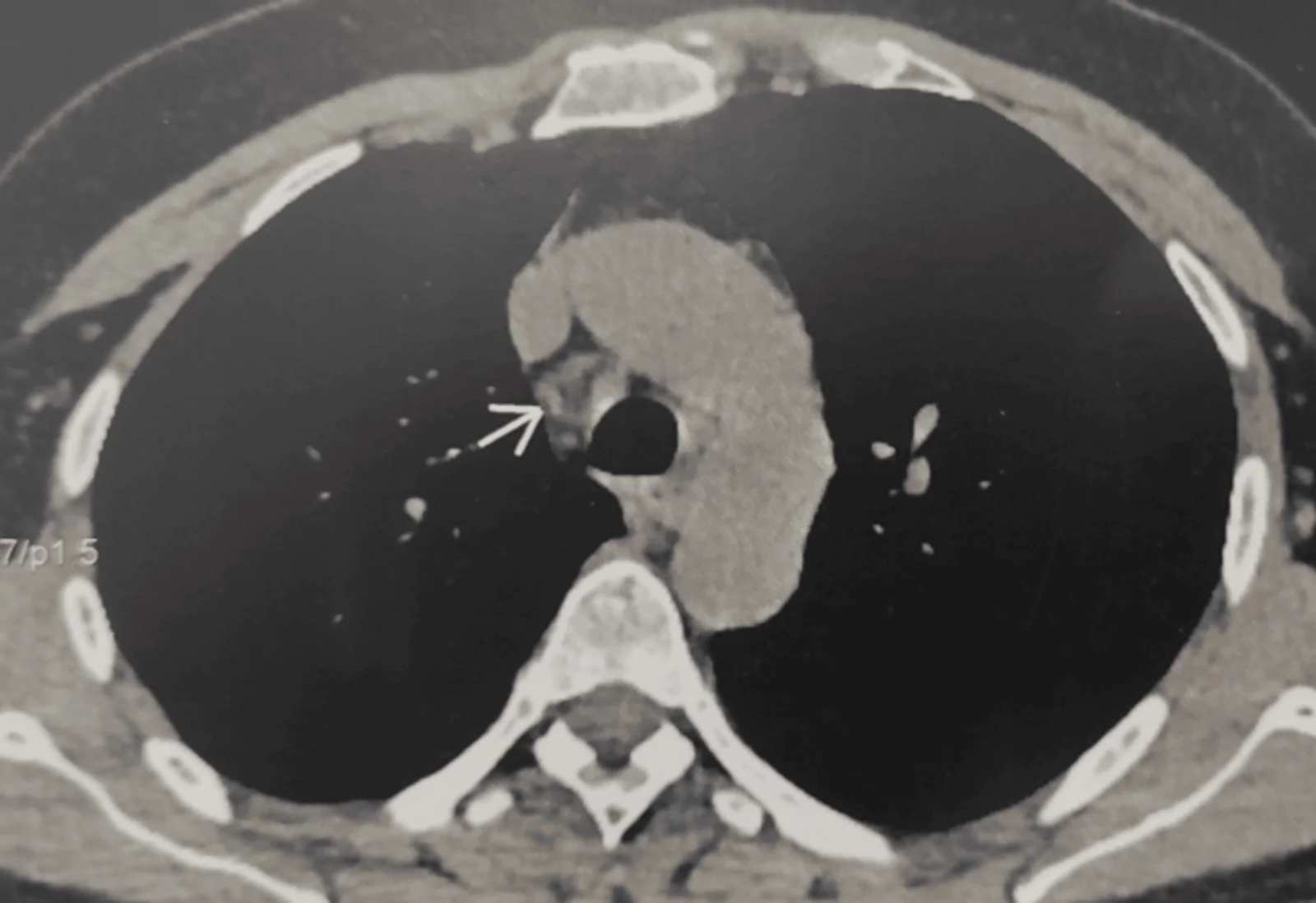
Follow-up chest computed tomography (CT) scan (2025, mediastinal window) Persistent mediastinal lymphadenopathy (white arrow)

A follow-up chest CT performed one year later showed complete resolution of the tree-in-bud micronodules on the lung parenchymal window (Figure 2). However, persistent mediastinal lymphadenopathy was observed on the mediastinal window (Figure 3), prompting further investigations.

**Figure 4:**
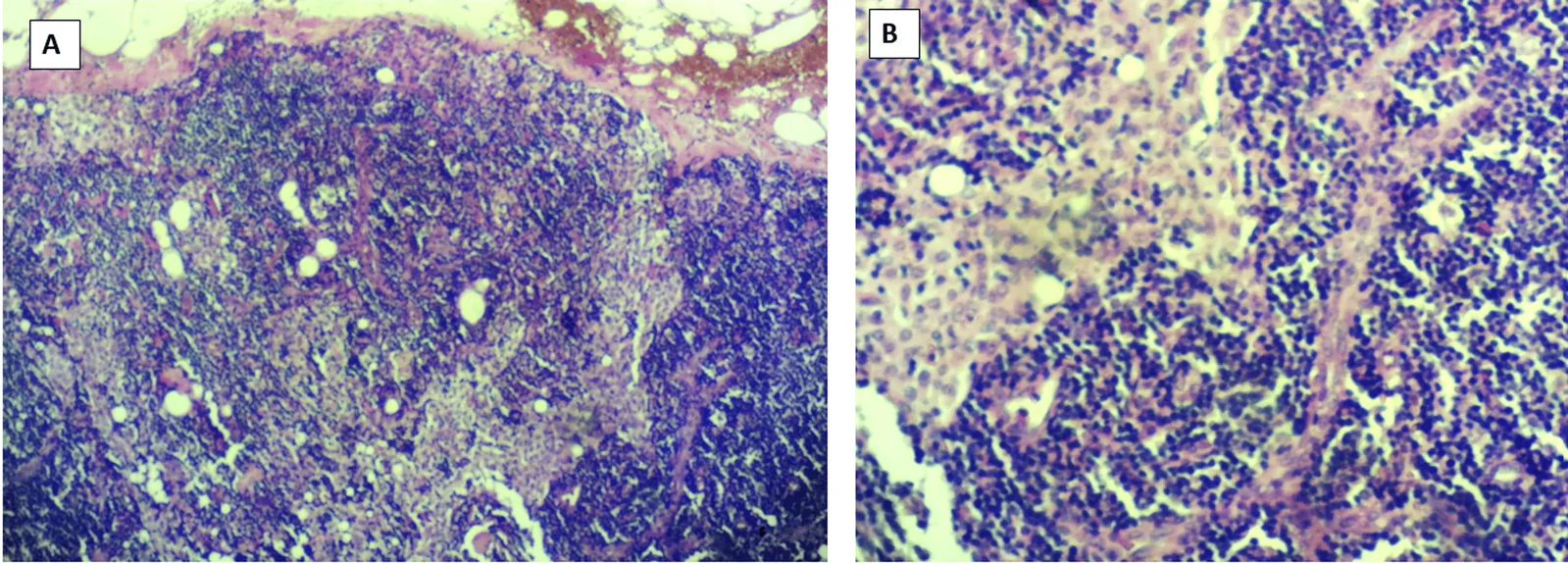
Histopathological examination of the excised axillary lymph node A. Hematoxylin and eosin (H&E, ×5) stain showing lymph node parenchyma with cortical reactive lymphoid follicles containing prominent germinal centers, associated with sinus histiocytosis. B. Higher-power view (H&E, ×10) highlighting sinus histiocytosis.

An excisional biopsy of an axillary lymph node demonstrated reactive follicular hyperplasia without evidence of malignancy or granulomatous inflammation (Figure 4). Xpert MTB/RIF performed on the biopsy specimen was negative.

Routine laboratory investigations, including complete blood count, liver and renal function tests, serum angiotensin-converting enzyme level, and calcium-phosphate metabolism, were all within normal limits (Table 1).

**Table 1:** Routine laboratory investigations at presentation

Parameter	Patient value	Reference range
Hemoglobin	13.4 g/dL	12.0-16.0 g/dL
White blood cell count	6.8 × 10⁹/L	4.0-10.0 × 10⁹/L
Platelet count	278 × 10⁹/L	150-400 × 10⁹/L
Serum creatinine	8.1 mg/L	6.0-12.0 mg/L
Serum angiotensin-converting enzyme (ACE)	42 U/L	8-52 U/L
Serum calcium	2.32 mmol/L	2.15-2.55 mmol/L
Serum phosphate	1.18 mmol/L	0.80-1.50 mmol/L

Flexible bronchoscopy revealed no endobronchial abnormalities. Histopathological examination of bronchial biopsies showed non-specific chronic inflammatory changes. Bronchoalveolar lavage was negative for *Mycobacterium tuberculosis* by Xpert MTB/RIF and culture, as well as for fungal, parasitological, and slow-growing bacterial investigations.

**Figure 5:**
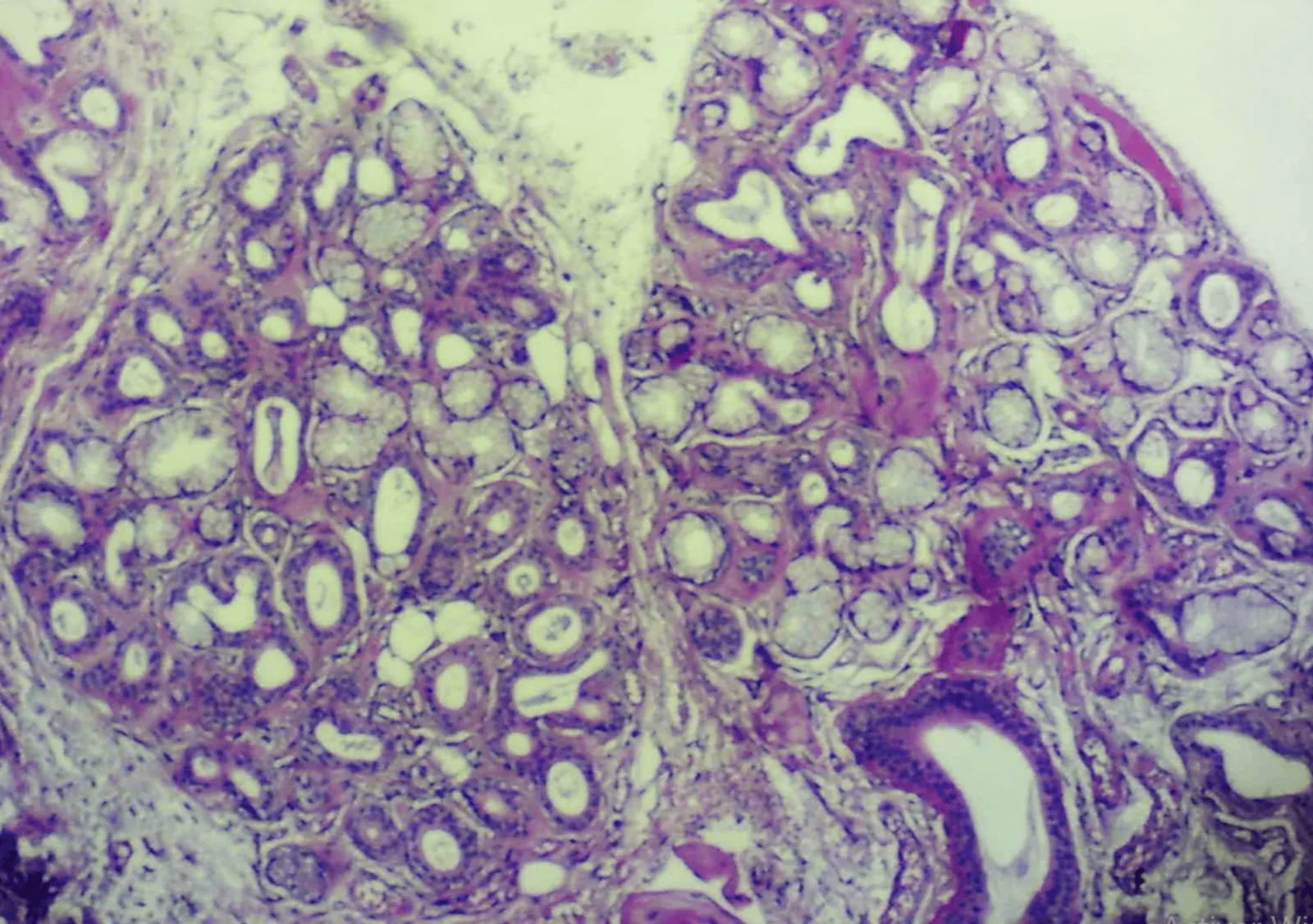
Histopathological examination of the minor salivary gland biopsy Hematoxylin and eosin (H&E, ×5) stain showing minor salivary gland tissue with fibrous stroma containing scattered lymphocytic inflammatory cells.

Further investigations, including minor salivary gland biopsy, ophthalmological examination, transthoracic echocardiography, pulmonary function tests, and bone marrow biopsy, failed to identify an alternative diagnosis. The salivary gland biopsy demonstrated non-specific chronic sialadenitis (Figure 5), whereas bone marrow examination showed reactive lymphoid infiltration without evidence of hematological malignancy.

Given the persistence of recurrent respiratory infections associated with unexplained lymphadenopathy despite an extensive negative diagnostic work-up, an underlying primary immunodeficiency was suspected. Serum protein electrophoresis revealed marked hypogammaglobulinemia (γ-globulin fraction: 6 g/L; reference range: 8-13 g/L). Quantitative immunoglobulin testing demonstrated profoundly decreased serum IgG levels (0.98 g/L; reference range: 7-16 g/L) with complete IgA deficiency (0 g/L; reference range: 0.7-4.0 g/L). Immunophenotyping showed normal T- and B-cell subsets. These findings were consistent with the diagnosis of CVID (Table 2).

**Table 2:** Immunological investigations leading to the diagnosis of common variable immunodeficiency

Parameter	Patient value	Reference range
Serum protein electrophoresis	Hypogammaglobulinemia (γ-globulin fraction: 6 g/L)	8-13 g/L*
Serum IgG	0.98 g/L	7.0-16.0 g/L
Serum IgA	0 g/L	0.70-4.00 g/L

After secondary causes of hypogammaglobulinemia had been excluded, the diagnosis of CVID was established. The patient was subsequently referred to the Internal Medicine Department for immunoglobulin replacement therapy and long-term follow-up.

## Discussion

CVID is the most common symptomatic primary immunodeficiency in adults and is characterized by hypogammaglobulinemia, impaired antibody production, and recurrent infections [1,2]. Because of its marked clinical heterogeneity, patients may present not only with recurrent infections but also with autoimmune, granulomatous, and lymphoproliferative manifestations, making the diagnosis particularly challenging and frequently delayed [3].

The present case illustrates this diagnostic challenge. Despite recurrent lower respiratory tract infections over one year, the persistence of mediastinal lymphadenopathy prompted extensive investigations for tuberculosis, sarcoidosis, lymphoma, and other infectious or inflammatory disorders. Histopathological examinations demonstrated only reactive changes, while microbiological investigations, including bronchoalveolar lavage and Xpert MTB/RIF, remained negative. The diagnosis was ultimately established only after serum protein electrophoresis revealed marked hypogammaglobulinemia.

Respiratory involvement is the leading cause of morbidity in patients with CVID and is reported in up to 90% of cases [4]. Recurrent pneumonia is the most common clinical presentation and may precede the diagnosis by several years. Chest CT findings are heterogeneous and include consolidations, bronchiectasis, pulmonary nodules, ground-glass opacities, and mediastinal lymphadenopathy [5]. In our patient, the initial tree-in-bud micronodules suggested an infectious bronchiolitis. However, follow-up imaging demonstrated complete resolution of the pulmonary micronodules while mediastinal lymphadenopathy persisted, supporting the need for further diagnostic evaluation beyond infectious causes.

Delayed diagnosis remains a major concern in CVID because recurrent respiratory infections are frequently attributed to common infectious diseases rather than to an underlying primary immunodeficiency. Several studies have shown that prolonged diagnostic delay is associated with irreversible pulmonary complications, particularly bronchiectasis and chronic lung disease [6]. Early recognition is therefore essential to reduce long-term morbidity.

Persistent lymphadenopathy represents a well-recognized non-infectious manifestation of CVID and reflects chronic immune dysregulation rather than active infection in many patients [7]. Histopathological examination usually reveals reactive follicular hyperplasia, as observed in our patient, although granulomatous inflammation may occasionally be present. Because these findings closely resemble lymphoma, tuberculosis, or sarcoidosis, many patients undergo repeated invasive investigations before CVID is considered [8].

Although CVID can occur at any age, diagnosis is most commonly established during the second to fourth decades of life. Nevertheless, the current European Society for Immunodeficiencies (ESID) diagnostic criteria recognize that the disease may also be diagnosed later in adulthood after exclusion of secondary causes of hypogammaglobulinemia [9]. Our patient illustrates that advanced age should not exclude the diagnosis when recurrent respiratory infections are associated with persistent unexplained lymphadenopathy.

The diagnosis of CVID relies on reduced serum IgG associated with low IgA and/or IgM concentrations, together with impaired antibody responses after exclusion of secondary causes of hypogammaglobulinemia [10]. In the present case, serum protein electrophoresis was the pivotal investigation that prompted quantitative immunoglobulin testing and established the diagnosis. This observation emphasizes the value of including serum protein electrophoresis early in the evaluation of adults with recurrent respiratory infections and persistent lymphadenopathy.

Immunoglobulin replacement therapy remains the cornerstone of CVID management and significantly reduces the frequency of infections, prevents progressive pulmonary damage, and improves long-term prognosis [11]. Consequently, early diagnosis not only avoids unnecessary investigations but also allows prompt initiation of appropriate treatment before irreversible complications develop.

This case highlights the importance of considering CVID in adults presenting with recurrent respiratory infections and persistent mediastinal lymphadenopathy after infectious, malignant, and granulomatous diseases have been excluded. Increased awareness of this uncommon presentation may shorten diagnostic delay, facilitate timely immunological evaluation, and ultimately improve patient outcomes [12].

## Conclusions

Delayed diagnosis of CVID remains a major clinical challenge because of its heterogeneous presentation and the absence of specific early manifestations. This case illustrates that recurrent pneumonia associated with persistent generalized lymphadenopathy may represent the initial presentation of CVID, even in patients without a previous history of immunodeficiency. When infectious, granulomatous, and malignant causes have been excluded, serum protein electrophoresis may serve as an inexpensive screening test and should prompt quantitative immunoglobulin measurement when hypogammaglobulinemia is suspected. Early recognition of CVID is essential to initiate immunoglobulin replacement therapy promptly, prevent irreversible pulmonary complications, and improve long-term outcomes.
